# Efficient and high-density immobilization of animal cells by a microfiber with both swelling and cell adhesion properties and its application to exosome production

**DOI:** 10.1007/s10529-025-03585-5

**Published:** 2025-04-12

**Authors:** Naofumi Shiomi, Pengfei Zhang, Shuji Nakatsuka, Kazuo Kumagai, Hideto Matsuyama

**Affiliations:** Research Center for Membrane and Film Technology, Kobe University, Kobe, Japan

**Keywords:** Electrospinning, Exosomes, High-density immobilization, Microfiber, Swelling

## Abstract

**Purpose:**

For high-density cell culture, we studied the development of optimal microfibers (MFs) with a 0.1–10 μm diameter, which due to their large surface area can serve as an immobilization carrier for animal cells. To date, few studies have used MFs as scaffolding for high-density cell culturing.

**Results:**

Using six types of nonsoluble synthetic polymers, MF sheets were fabricated by electrospinning. The cellulose acetate, polyketone, and polyvinyl acetate MFs exhibited swelling and water retention capacities. Next, the six types of MF fragments were examined for immobilizing TKD2 mouse vascular endothelial cells. Although most cells were taken into the three MFs characterized by swelling, most leaked from the MFs without adhesion. To solve this, the MF sheets comprising cellulose acetate and polyketones were coated with gelatin. Although the adhesive capacity was enhanced, the swelling capacity decreased and almost all the immobilized mouse cells remained on the sheets’ surfaces. Based on these results, we produced a novel MF sheet comprising a gelatin, cellulose acetate, and polyketone mixture (CPG). Since the cells were taken into the MFs by swelling and attached by the gelatin, the CPG fragment immobilized almost all the supplied cells with little loss and reached a high density of 3.2 × 10^9^ MF-g^−1^, Furthermore, the immobilized cells continuously produced exosomes with a high productivity of 6–7 × 10^10^ particles ml^−1^ after either 8 h or 16 h of culturing.

**Conclusion:**

CPG-based MFs are expected to have a wide range of future applications, including exosome production from animal cells.

## Introduction

Exosomes are endosomes formed inside cells that are secreted to communicate with other organs via proteins and RNAs (Hood et al. 2009; Li et al. 2018). Recently, exosomes have emerged as reliable biomarkers for diagnosing many diseases, such as tumors and metabolic diseases, and have therapeutic effects on anti-inflammatory atopic dermatitis, anti-aging effects, immune activation, and suppression through immunomodulatory effects (Yang et al. 2021; Liu et al. 2022; Malekian et al. 2023; Chen et al. 2024). Exosomes have the following advantages: (1) lower ethical barriers than regenerative medicine, (2) low immunogenicity, even for exosomes from other cell families, and (3) easy sterilization and storage; they are currently expected to replace cell-based regenerative medicine.

However, several problems are associated with the therapeutic use of exosomes. Exosomes contain less than 1 μg of protein per 1 ml of culture broth of animal cells, but the amount of pharmacologically active exosomes is thought to be around 100–500 μg of exosome protein in a mouse, and therefore, a huge amount of exosome proteins is required for human therapy (Yi et al. 2020; de Almeida Fuzeta et al. 2020). Additionally, an exosome production technology has not been sufficiently established. Moreover, because normal somatic cells have a division limit and differentiated somatic cells other than stem cells are not proliferative the mass production of exosomes is even more difficult. Therefore, it is desirable to develop a continuous production process using densely immobilized animal cells. Several useful methods using microcarriers and hollow fibers have already been developed for cell mass culture (Kimiz-Gebologlu and Oncel 2022); however, further improvement is necessary because the cell densities in these immobilization processes are still inadequate for exosome production. Meanwhile, few studies have been conducted on mass cell culture using microfibers and nanofibers (Reddy et al. 2021; Venmathi Maran et al. 2024), even though their surface areas and voids are larger than those of microcarriers and hollow fibers, making them promising for high-density cell immobilization.

Thus, in this study, we attempted to develop the best submicron-size microfiber (hereafter MF) for high-density cell culture by adding both hydrophilicity and cell adhesion properties. Consequently, we found that MFs fabricated from cellulose diacetate (CA) have high swelling properties and are suitable for uptaking cells into MFs; polyketone (PK) provided MFs flexibility, and a novel MF made of a CA, PK, and gelatin (GEL) blended polymer was produced. When the cell suspension was added to this MF, almost all supplied cells were taken into the MF and could be immobilized within 6–8 h without cell leakage, and after 10–15 days of culturing could reach a very high density per MF. Furthermore, cells immobilized in CPG-based MF were extremely productive, continuously producing exosomes for a long time.

## Materials and methods

### Chemicals

Polylactic acid (PLA) (Nature3D, Tokyo, Japan), polystylene (PS) (182427 - 25G; Sigma-Aldrich, St. Louis, Missouri), poly(acrylonitrile-co-butadiene) (NBR) (REF180912 - 100G;Sigma-Aldrich,), poly(ethylene-co-vinyl acetate (EVA) (340502 - 250G; Sigma-Aldrich), poly(ethylene-co-vinyl alcohol)(EVOH)(414093 - 100G; Sigma-Aldrich), poly(ethylene oxide) (PEO) (REF182028 - 250G; Sigma-Aldrich), CA (L44, Daicel Corp., Osaka, Japan), PK (#360, Asahikasei Corp., Tokyo, Japan) were used as synthetic polymers. Gelatin (GEL) from bovine bone (REF071 - 06291; FUJIFILM Wako Pure Chem. Corp., Osaka, Japan), sodium alginate 80–120 (ARG) (194-13321, FUJIFILM Wako Pure Chem. Corp.) were used as the natural polymers. N,N-dimethyl formamide(DMAc), acetone, ethanol(EtOH), dimethyl sulfoxide (DMSO), 1,1,1,3,3,3- hexafluoroisopropanol (HFP) were used as solvents to dissolve the polymers. StemSure 0.1% w/v Gelatin Solution (REF19015805; FUJIFILM Wako Pure Chemicals Corp.) was used to coat the MFs.

### Cell and media

Mouse TKD2 vascular endothelial cells (RRID: CVCL_5598) were obtained from the JCRB Cell Bank (IFO 50374). Modified Eagle's medium (MP Biomedicals Inc., Illkirch-Graffenstaden, France) was mixed with fetal bovine serum (FBS)(26140 - 079; ThermoFisher Scientific, Waltham, MA, USA) at a ratio of 9:1 and a 1/100 volume of penicillin/streptomycin solution (REF 168231 - 9; Fujifilm Wako Pure Chemicals Corporation) was added and used as the basic medium (hereinafter referred to as DMEM). A 1:9 mixture of FBS without exosomes (Exo-FBS- 50 A- 1; System Biosciences, Palo Alto, CA, USA) and DMEM (hereafter referred to as ExoFBS-DMEM), and EV-Up MSC EV production (REF053 - 09451; FUJIFILM Wako Pure Chem. Corp., hereafter referred to as EV-Up medium) was used for exosome production. For cell detachment, 2.5% trypsin solution (15090 - 046; ThermoFisher Scientific, Waltham, MASA,) was used, and phosphate-buffered saline (PBS) was used for washing. Cells precultured for at least 1 week were used and cultured statically in a CO_2_ incubator at 37 °C.

### Preparation of MFs

Most of MFs were prepared according to previously described methods (Tungprapa et al. 2007; Bonino et al. 2011; Aoki et al. 2015; Zhang and Chase 2016; Yamashita et al. 2018; Uyar and Besenbacher 2008; Dou et al. 2021; Unverzagt et al. 2024). PS (20 wt%) was dissolved in DMF, PLA (6 wt%) was dissolved in DMAc/EtOH (9:1), PK (5 wt%), and GEL (3 wt%) were dissolved in HFP, NBR (6 wt%) was dissolved in acetone, EVA (16 wt%) was dissolved in chloroform, EVOH (12 wt%) was dissolved in HFP, CA (15 wt%) was dissolved in a solvent with a 3:1 acetone:DMAc weight ratio, ARG (13.5 wt%), PEO (4 wt%), Triton-X (1%) were dissolved in distilled water. The solution was placed in a 20 ml plastic syringe (SS- 20ESPZ, Terumo Co., Tokyo, Japan) using a 1.20 × 38 mm syringe needle and electrospinning was performed using an electrospinning device (NEU KATO TECH Co., Ltd.) under the conditions shown in Table 1.

**Table 1:** Conditions of electrospinning

Polymer	Distance^*1^ (cm)	Target rate^*2^ (m min^−1^)	Traverse rate^*3^ (m min^−1^)	Feed rate (ml min^−1^)	DC power (kV)
PLA	15	0.3	4	0.13	20
PK	15	0.3	5	0.13	20
CA	15	0.3	5	0.13	25
PS	15	0.2	5	0.5	20
NBR	15	0.2	5	0.25	20
EVA	15	0.3	5	0.15	25
EVOH	15	0.4	5	0.23	20
GEL	15	0.4	5	0.25	20
ARG	15	0.4	5	0.1	25
CPG	15	0.4	5	0.2	20

* 1: Tip-to-collector distance, * 2: Rotation speed of the drum for spinning the polymer, * 3: Syringe transfer speed

### Shape and hydrophilicity of MFs

Fragments of the MF sheet were coated with platinum using a platinum coating device (JEOL, Tokyo, Japan, JFC- 1600) at 20 mV for 60 s and observed using a scanning electron microscope (SEM) (Phenom ProX G6; Thermo Fisher Scientific). The diameters of the MFs were measured from the SEM images using ImageJ software, and the average value was calculated. The MFs’ thicknesses were measured using a thickness-measuring instrument (Peacock model H; OZAKI MFG. CO., LTD., Tokyo, Japan). The static contact angle (initial contact angle at 0.1 ms) and absorption rate of the droplet into the MF were measured using a solid–liquid interface analyzer (DropMaster 300; Kowa Interface Science Co. Ltd., Saitama, Japan). The moisture retention rate of MF was determined by weighing 4–8 mg of MF fragments in a 1.5 ml Eppendorf tube after being moistened for 1 h and then removed and left for 10 min.

### Culture of vascular endothelial cells using a culture dish and adhesion of cells to films

TKD2 vascular endothelial cells (5 × 10^4^ cells per dish) were seeded in 16 dishes (Eppendorf, Hamburg, Germany; REF:0030700015) containing 2 ml of DMEM. Two dishes were taken from a CO_2_ incubator every day. The medium was removed and washed once with PBS. The cells were stripped with 2.5% trypsin solution and centrifuged (1000×*g*, 3 min) to collect them. The number of cells was counted using a cell counter (WC2 - 100; WAKEN Tech Co., Ltd., Kyoto, Japan) after suspension in PBS. The glucose concentration in the medium was determined using a glucose kit (Glucose CII Test Wako; FUJIFILM Wako Pure Chemical Co.), and the absorbance was measured at 450 nm using a microplate reader (INFINITE M; PLEX, Tecan Japan CO., Ltd., Tokyo, Japan).

The cells’ adhesion to the polymeric films was measured using the following method: A 5 wt% polymer solution was prepared by dissolving the polymer in the same solvent used for MF preparation, and 1 mL of the solution was placed in a 30 mm diameter glass Petri dish (82-1681; Sansho Co., Tokyo, Japan), and the solvent was slowly evaporated to prepare thin films. The resulting films were sterilized by autoclaving at 120 °C for 20 min (70% ethanol for EVOH films). A 30 mm culture dish (0030700015; Eppendorf) was used as a positive control. Mouse TKD2 cells (5 × 10^4^ cells per dish) were added to those dishes coated by the films containing 2 ml of DMEM, cultured for four days, and washed three times with PBS. The cells were then stripped from the film with 0.5 ml of 1% trypsin solution, and the cells were counted using a cell counter (WC2 - 100).

### Immobilization of TKD2 cells on MFs

Experiments on the immobilization of cells in MFs made of six synthetic polymers (PLA, PK, CA, PS, EVOH, and EVA) were performed as follows: MF sheets were cut into 4.0 mg (approximately 4–6 mm^2^ in size) (hereinafter referred to as MF fragments). These MF fragments were placed in 1.5 ml microtubes and sterilized by autoclaving at 120 °C for 15 min. For EVA, 8.0 mg were sterilized in 70% ethanol for 4 h, washed with sterile water, and dried. Cell uptake and adhesion were performed by adding 50 μl of cell suspension (1 × 10^5^ cells) to the MF fragments and incubating them for 6 h, followed by the addition of 50 μl of DMEM for 12 h. The MF fragments were then removed using tweezers and placed in a 48-well plate (1830 - 048, IWAKI& Co., Ltd., Tokyo, Japan), which was used for the suspended cells. The number of cells and volume of fluid remaining in a 1.5-ml microtube were measured to determine the amount of uptake into the MF fragments. After 1 d of incubation in a 48-well plate, the MF fragments were removed with tweezers and placed in another well of a 48-well plate, and 0.9 mL of DMEM was added to start the culture. Thereafter, the medium was changed every 3 days when the cell count was 1 × 10^5^ or less and every 2 days when the cell count was higher.

The number of viable cells immobilized on MF fragments was determined with Cell Counting Kit 8 (341-07761; Fujifilm Wako Pure Chemicals); 300 μl of DMEM and 15 μl of WST- 8 solution were added to each well containing MF fragments and incubated in a CO_2_ incubator at 37 °C with stirring every 3 min. After a certain period, 50 μl was sampled and placed in a 96-well plate (6052630; Areaspectraplate) and absorbance at 450 nm was measured by a microplate reader (INFINITE M PLEX). The number of viable cells was calculated using a calibration curve previously used to examine the relationship between cell number and WST- 8 activity. After the cell activity measurements, fresh DMEM was added, and the cells were cultured.

Cells immobilized on the MF fragments of PLA were subjected to the following pretreatment and observed by SEM: MF fragments immobilized for 15 days were placed in 24-well plates, washed once with PBS, and immobilized with 2.5% glutaraldehyde solution for 30 min. The MF fragments were then dehydrated in 2 ml of 50%, 70%, 80%, 90%, and 100% ethanol solutions, replaced twice with t-butyl alcohol, and lyophilized. The resulting samples were platinum-coated and observed using SEM.

### Immobilization of cells on gelatin-coated MFs

PK and CA MF fragments (4.0 mg) were sterilized by autoclaving in 1.5 ml Eppendorf tubes at 120 °C for 20 min. MF fragments were suspended in 500 μl of StemSure solution and incubated for 16 h to coat the MF fragments with GEL. After the GEL-coated MF fragments were taken out and placed in a 48-well plate (1830 - 048; IWAKI & CO., LTD.), 100 μl of DMEM medium solution containing 1 × 10^5^ cells was added and left for 6 h. Then 50 μl of DMEM was gently added and incubated for 12 h. The MF fragments were removed and placed in a new well of a 48-well plate and 0.9 ml of DMEM medium was added to the culture. The medium was changed every two days. The number of cells immobilized on the MF fragments was determined using a Cell Counting Kit 8. Cells immobilized on the MF fragments were pretreated in the same manner as PLA and observed using SEM. Exosomes were produced using immobilized cells as follows: TKD2 cells were cultured and immobilized in three gelatin-coated CK- or CA MF fragments for 20 days described above and three fragments were placed in a well of 24-well plate. Then 1.5 ml of EV-up medium was added and cultured for 2 days, and the medium was exchanged. After the operation was repeated three times, the medium was changed to 1.5 ml ExoFBS-DMEM medium, and a similar batch operation was repeated three times.

### Purification of exosomes and nanoparticle tracking analysis

The exosomes from the culture broth were purified according to the method provided by the Merck Corporation: The culture broth was centrifuged at 2000×*g* for 3 min, and the supernatant was filtered through a 0.20 μm sterile filter (REF:S- 1302; Kurabo corp., Osaka, Japan). The filtrate was placed in a filtration filter (Amicon Ultra- 0.5 ml Centrifugal Filters UFC501096; Merck, Darmstadt, Germany) that fractionated the filtrate at 10,000 molecular weight and centrifuged it at 10,000×*g* for 10 min. To wash the exosome solution, a PBS solution passing through the filtration filter at 30,000 molecular weight (Amicon Ultra- 0.5 ml Centrifugal filters; Merck, Darmstadt, Germany) was used to wash the exosome solution. The exosome solution was obtained by adding 500 μl of the PBS solution to a filter, centrifuging it at 10,000×*g* for 10 min, adding 250 μl of PBS solution and suspended. The obtained exosome solution was diluted to 1/20–1/100 with ultrapure water, and the nanoparticles tracking analysis (NTA) was immediately performed using a nanoparticle-measuring device (NanoSight NS500-HSBZ-K, Quantum Design, Tokyo, Japan).

### Novel nanofiber showing swellability and strong cell adhesion characteristics

Novel nanofiber was produced by the following method: A solution of CA and PK dissolved in HFP and a solution of GEL dissolved in HFP were mixed to obtain a solution comprising 1.76 wt% CA, 1.29 wt% PK, and 1.05 wt% GEL (hereafter this blended polymer is referred to as CPG). The MF was then produced under the conditions described in Table 1. SEM images of the CPG-based MF were obtained as previously described, and the transmittance of the MF was measured using an FT-IR spectrometer (Nicolet iS5; Thermo Fisher Scientific). Two MF sheets with different film thicknesses (0.6 and 0.25 mm) were produced.

The cells were immobilized on a CPG MF fragment using the methods already described: A solution of TKD2 cells (1 × 10^5^ cells) suspended in 100 μl and 30 μl of DMEM were dripped onto the thicker CPG MF fragment (4.0 mg in weight,) and the thinner CPG MF fragment (0.3 mg in weight), respectively and incubated for 6 h. 50 μl of DMEM was gently added followed by incubation for another 12 h. These MF fragments were then transferred to 48-well plates (1830 - 04), and cells within the MF fragments were grown in the MFs by replacing 0.9 ml of DMEM every 2 days. The number of immobilized cells was determined by measuring the mitochondrial activity using WST- 8. Cells immobilized on the MF fragments were pretreated similarly to the PLA version and observed using SEM. The volume of MF fragment containing immobilized cells was calculated as follows: a 1.5-ml microtube containing two fragments was filled up to 1 ml with water, which was then removed from the tube, and the mass was weighed.

Three CPG MF fragments cultured for 20 days were placed in a 24-well plate, 0.5 ml of ExoFBS-DMEM per 4 mg of MF fragments was added, cultured for 2 days, and the medium was replaced. After this operation was repeated eight times, two fragments were added to a 24-well plate (total 5 fragments), 0.25 ml of medium per 4 mg MF fragments was added, cultured for one day, and the medium was replaced. After this operation was repeated eight times, 0.125 ml of medium per 4 mg of MF fragments was added, the cells were cultured for 16 h and 8 h (total 24 h) and the medium was replaced. This procedure was repeated 4 times. The exosomes were purified in the medium, and the number of exosomes was measured as described above.

### Statistical analysis

Multiple independent experiments were performed for each test, and the mean and standard deviation (SD) were calculated. Independent experiments number (n) and mean ± SD were shown in figure capture.

## Results

### Hydrophilicity and moisture retention capacity of MF sheets

**Fig. 1:**
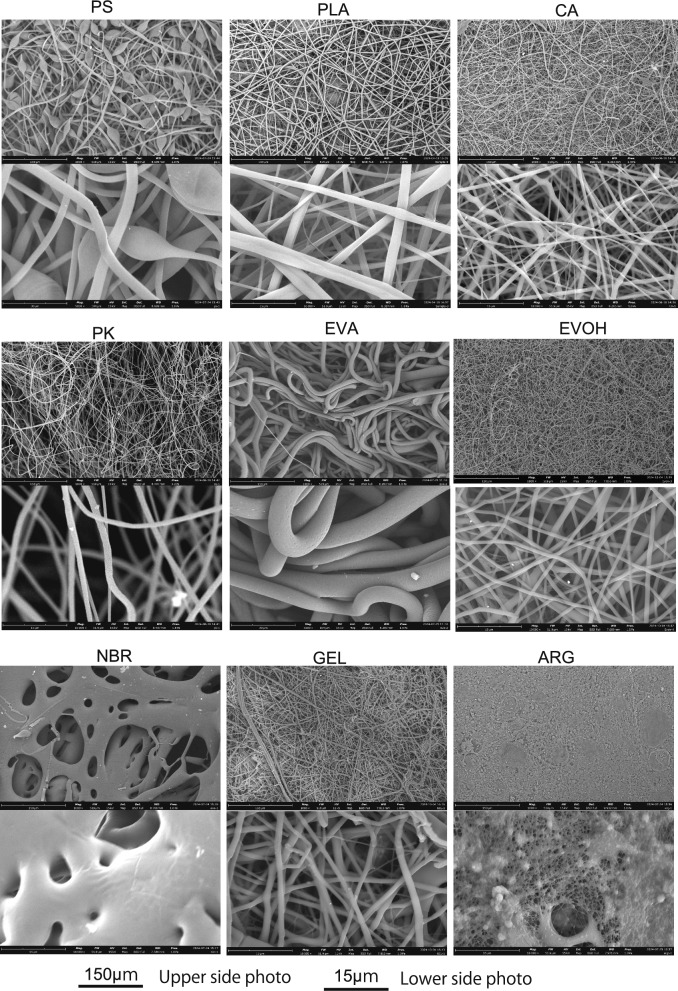
SEM images of MFs

**Fig. 2:**
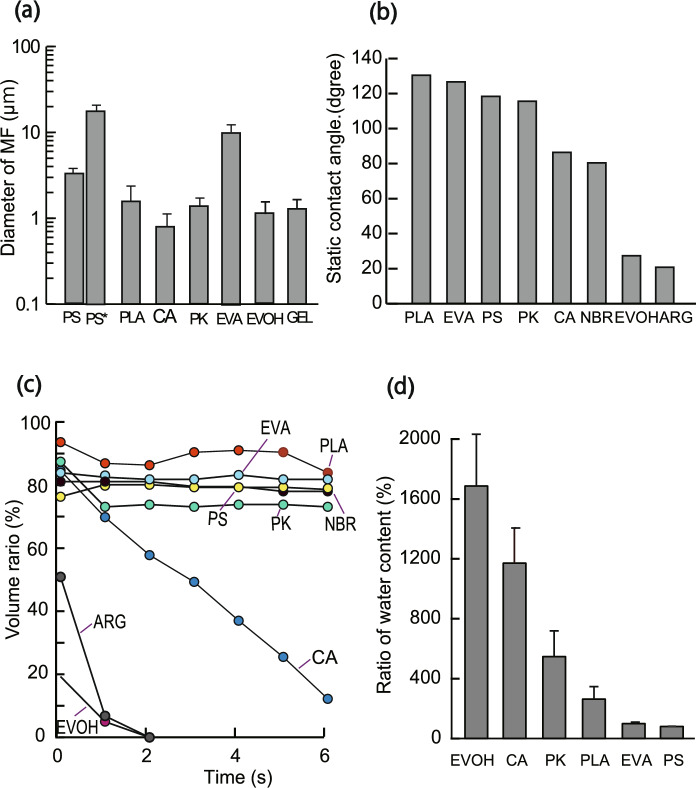
Hydrophilic properties of MF sheets. **a** Average diameter of MFs. PS* is the spherical portion of the PS MF (n = 50, mean ± SD). **b** Static contact angles (initial contact angle at 0.1 ms) of MFs after droplet drop. **c** Percentage of the volume of water retained in the MF sheet without uptake relative to the volume of the initial droplet, when a droplet was dripped to the MF sheet. **d** Water retention ratio relative to MF mass (n = 3, mean ± SD)

Immobilizing carriers for continuous animal cell culture is necessary to ensure cost efficiency, non-degradability, low cytotoxicity, and water insolubility. Thus, seven types of synthetic polymers (PS, PLA, PK, CA, NBR, EVA, and EVOH) with these characteristics were selected, and MF sheets were produced using an electrospinning apparatus. For comparison, MFs comprising the water-soluble natural polymers ARG containing PEO and GEL were prepared. Figure 1 shows a SEM image of the obtained MF sheets, and Fig. 2a shows the average diameter of the fibers calculated from the SEM images. Fibers fabricated from five synthetic polymers (PS, PLA, PK, CA, EVOH) and one water-soluble polymer (GEL) had a diameter of 0.8–1.3 μm, while EVA, with its rubber properties, had a very large diameter (20 μm). The ARG MF was a mixture of very fine nanofibers and electrospray. Because the NBR MF did not adopt the fiber structure, it was not used in the subsequent immobilization tests. The average thickness of the MFs fabricated from six synthetic polymers except NBR was 0.40–0.65 mm. Fibers larger than 50 μm are called microfibers, whereas those smaller than 100 nm are called nanofibers. The fiber fabricated in this study is between these two sizes, approximately submicron-sized fibers, but will be referred to here as MFs.

To immobilize animal cells deep inside MFs, an aqueous solution containing the cells must be placed into the hydrophobic MF. Therefore, the balance between the hydrophilicity and hydrophobicity of MF is critical. The results of the static contact angle (initial contact angle at 0.1 ms) and time-related changes in water droplet absorption into the MF sheet are shown in Fig. 2b and c, respectively. The higher the hydrophobicity of the MF surface, the larger in the static contact angle, and the higher the hydrophobicity of the MF interior, the lower the rate at which water droplets are taken up. The EVOH-fabricated MF sheets were highly hydrophilic and water droplets were taken up immediately, the CA and PK MF sheets took up water droplets slowly, and the PLA-fabricated MF sheet was much more hydrophobic than hydrophilic, and only a small amount of water was taken up. In contrast, the PS- and EVA-based MF sheets were highly hydrophobic, and water droplets were hardly taken up. Moreover, the water take-up and retention of the MF sheets fabricated from the six synthetic polymers (PLA, PK, CA, PS, EVA, and EVOH) were examined, and Fig. 2d shows the results of the water retention properties of the MF sheets. PS and EVA made MF sheets hardly swell at all, and the PLA MF sheets swell only slightly. The CA-, PK-, and EVOH-fabricated MF sheets exhibited high water retention capacities. These results suggest that, although film and particles made of CA, PK, and EVOH are hydrophobic, they developed both water incorporation and water retention characteristics when the MF structure was formed.

### Immobilization characteristics of vascular endothelial cells to thin films and NF fragments

**Fig. 3:**
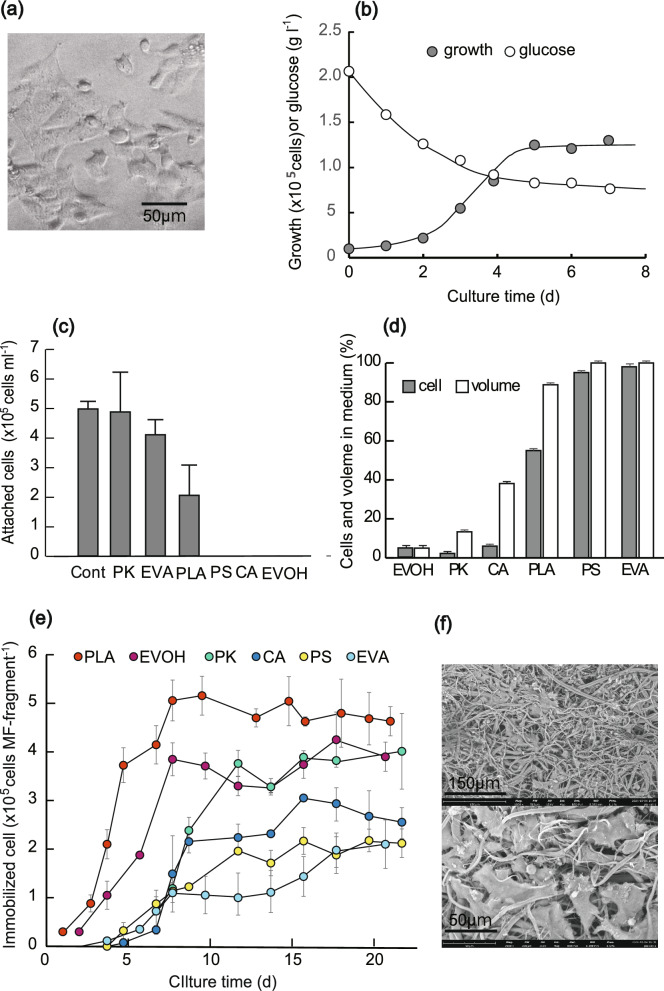
Immobilization characteristics of TKD2 cells to dishes, films, and MFs. **a** Image of TKD2 cells cultured on a dish. **b** Average growth per dish and glucose consumption in the medium when TKD2 cells were cultured on dishes (n = 2). **c** Cell number attached films made of synthetic polymers when TKD2 cells were cultured for 4 days (n = 3, mean ± SD). **d** The number of cells that were not taken into the MF fragments and the amount of rest medium when cell suspensions (1 × 10^5^ cells) were added into microtubes containing MF fragments (4.0 mg) and incubated for 16 h. **e** Change over time in the number of immobilized cells when TKD2 cells were cultured with 4 mg of MF fragments for 22 days. The number of inoculated cells is 1 × 10^5^. (mean ± SD, n = 3), and the number of immobilized cells was determined with Cell Counting Kit 8. **f** SEM images of immobilized TKD2 cells in PLA MF made (15 th day culture)

The adhesion capacities of the films and MFs to animal cells were compared with those of culturing TKD2 cells in a culture dish. TKD2 vascular endothelial cells (Fig. 3a) were used because exosomes from vascular endothelial cells have recently been used as therapeutic agents for heart disease. The results are shown in Fig. 3b. The cells adhered to the culture dish after several hours and started proliferating on the first day of culture, indicating that TKD2 cells were able to adhere and rapidly proliferate well when the culture dish was used as a scaffold. Second, cells were inoculated into glass dishes coated with thin films of polymers and cultured for 5 days, the results of which are shown in Fig. 3c. The PK, EVA, and PLA films had high cell adhesion capacity and were similar to the culture dish (control), while the PS, CA, and EVOH films did not attach.

Next, the cell immobilization capacities of the MF fragments fabricated from the PS, PLA, CA, PK, EVA, and EVOH polymers were examined. Cell suspensions (1 × 10^5^ cells) were added to microtubes containing the MF fragments (4.0 mg) and incubated for 16 h. The percentage of cells that did not enter the MF fragments and the amount of medium remaining are shown in Fig. 3d. The PK, CA, and EVOH MF fragments, which showed hydrophilic characteristics, swelled and absorbed the cells and medium, taking up > 90% (cells) and 60% (medium) into the MF fragments, whereas the PS and EVA MF fragments, which are highly hydrophobic, hardly incorporated the cells into the MF fragments. The PLA MF, which is rather hydrophobic, swelled slowly, and approximately 40% of the inoculated cells were immobilized therein. This result supports the hydrophilicity results shown in Fig. 2c and d.

The cells placed with the MFs in Fig. 3d were subsequently cultured, and the medium was changed. The number of cells immobilized on the MF fragments is shown in Fig. 3e. In the case of the PK, CA, and EVOH fragments, even though most of the inoculated cells (1 × 10^5^ cells) were taken in, they drastically decreased once and then began slowly to proliferate; the cells in the EVOH MF fragment decreased less than those of CA and PK, but still initially decreased by approximately 90%. This may have been due to the MFs inability to adhere to the uptaken cells, resulting in cell leakage when the medium was exchanged. Meanwhile, since the initial cell uptake of the PS- and EVA-based MF fragments was low, their growth was severely inhibited. The cells grew well using the PLA MF fragment, and the growth rate was the highest among the six types of MFs. This may have been due to the high adhesion force, although the swelling capacity was much lower than those of the PK, CA, and EVOH MF fragments. Figure 3f shows SEM images of the PLA MF after 15 days of immobilization, showing that the cells were well immobilized.

### Immobilization of cells with GEL-coated MF fragments

The PK and CA MF fragments leaked out most of the cells taken into the MF, although their capacity for incorporating cells into the MF by swelling was excellent. Thus, we attempted to increase the adhesion ability of the cells by coating them with GEL. After the MF fragments were coated with 0.1% GEL (Stemsure solution), cell suspensions were added to the PK and CA MF fragments, incubated for 8 h, and subsequently cultured using a method similar to that shown in Fig. 3d.

**Fig. 4:**
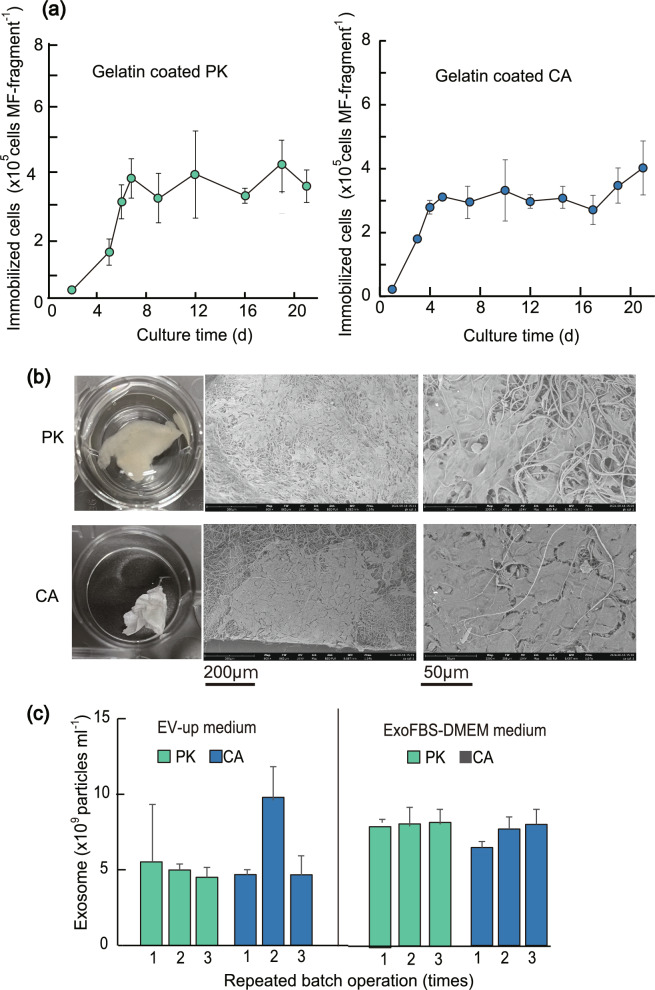
Immobilization of cells with GEL-coated MF fragments. **a** Change over time in the number of immobilized cells when TKD2 cells were cultured with 4 mg of GEL-coated MF fragments made of CA and PK for 22 days. The number of inoculated cells is 1 × 10^5^ and the number of immobilized cells was determined with Cell Counting Kit 8. (mean ± SD, n = 3). **b** Images of MFs in 48 well plate and SEM images of immobilized TKD2 cells in GEL-coated PK- and CA MFs. (15 day culture). **c** Numbers of exosome particles per medium when TKD2 cells were immobilized in GEL-coated PK and CA MFs (4 mg) were respectively cultured in 0.5 ml of EV-up and ExoFBS-DMEM media for 2 days. The operation was repeated three times. (mean ± SD, n = 3)

Figure 4a shows the number of immobilized cells. The MF fragments were already swollen with water by the GEL-coating operation, and the absorption capacity of inoculated cells was much lower than that of the MF without coating, causing inoculated cells to be lost. However, the absorbed cells grew much better than those without the GEL coating (Fig. 3e), and the cell density immobilized with PK and CA MF fragments reached their maximum values on the 7 th and 5 th days of culture. An SEM image of cells on the 15 th day of the culture is shown in Fig. 4b. The cells grew while adhering to the surface of the gel-coated MFs, and there were considerable areas inside the MFs where the cells were not immobilized.

Next, we examined whether the continuous production of exosomes was possible using immobilized cells 20 days after immobilization. Figure 4c shows the productivity of exosomes when three consecutive batch cultures were performed using ExoFBS-DMEM and EV-Up media. Exosomes could be continuously produced by the immobilized cells, and the productivity of exosomes per medium in ExoFBS-DMEM was much higher than that in the EV-Up medium, with an average production of 8.1 × 10^9^ and 7.4 × 10^9^ exosome particles ml^−1^ for 2 days of culture in PK and CA MF fragments (12 mg), respectively.

### Preparation of novel nanofibers showing swelling and strong cell-adhesion properties

**Fig. 5:**
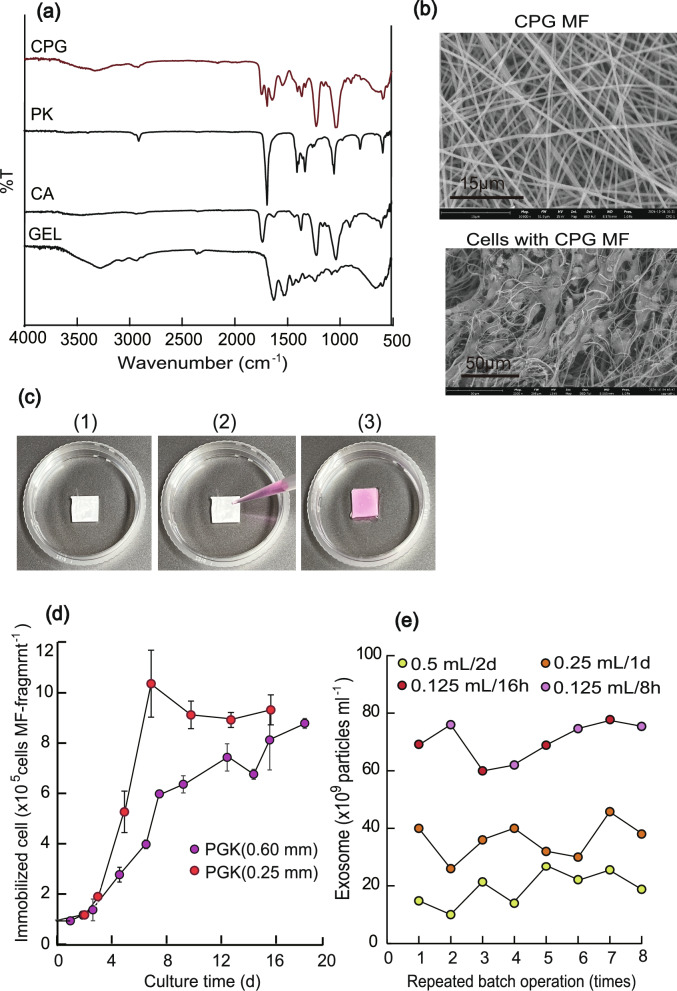
Immobilization of cells with CPG MF fragment. **a** Infrared spectra of CPG, PK, CA, and GEL MFs. **b** SEM images of CPG MF (upper side photo) and immobilized cells in CPG MF (12 day culture). **c** Procedure for immobilizing cells in CPG MF fragments. (1) MF fragment. (2) Drop cell suspension in the MF fragment. (3) Cultured MF swollen with cell suspension for over 6 h. **d** Change over time in the number of immobilized cells when TKD2 cells were cultured with 4 mg (0.60 mm thickness) and 0.3 mg (0.25 mm thickness) of CPG MF fragments. The number of inoculated cells is 1 × 10^5^ and the number of immobilized cells was determined with Cell Counting Kit 8 (mean ± SD, n = 3). **e** Numbers of exosome particles per medium when TKD2 cells immobilized in MFs were cultured in Exo-FBS-DMEM media. Three different conditions were applied: (i) 0.5 ml of medium per fragment and incubated for 2 days, (ii) 0.25 ml of medium per fragment and incubated for 1 day, and (iii) 0.125 ml of medium per fragment and incubated for 16 h and 8 h. The operation was repeated eight times for each condition

When coated with GEL solution, there were two problems: (1) cells bound only to the surface of the MF fragments, and (2) the MFs were already swollen. To overcome these drawbacks, we prepared an improved MFs from a mixture of GEL, PK and CA polymers, showing swelling and strong cell-adhesion properties (hereafter, this polymer mixture is abbreviated as CPG). In the CPG MF, CA was responsible for swelling, PK for hydrophilicity and softness, and GEL for strong cell adhesion capacity. Figure 5a shows FT-IR spectrum of CPG and the upper side image of Fig. 5b shows a SEM image of the CPG MFs, suggesting it was approximately uniform with an average diameter of 0.70 μm, suggesting that CA, PK, and GEL was apparently blended. Two CPG MFs of different thicknesses (0.60 mm and 0.25 mm) were prepared and used in the following experiments.

Figure 5c shows the swollen appearance of the CPG MF (0.60 mm thick); when the cell suspension was dropped, it was rapidly absorbed, and the cells were attached to the MF fragments after 4–8 h of incubation**.** The purple circles in Fig. 5d show the number of cells immobilized using CPG MF (0.60 mm in thickness) by an operation similar to that shown in Fig. 4a. Unlike the other MF fragments (Figs. 3e and 4a), the inoculated cells grew well with little leakage, and the final cell density was the highest. The lower side of Fig. 5b shows a SEM image of the cells at 12 days after immobilization, showing that the cells penetrated deep into the interior of the MF and proliferated. However, cell-free areas remained inside the MF, suggesting that the thickness of the MF and the initial supplied cell volume were suboptimal. Thus, a similar experiment was performed by increasing the number of supplied cells per MF ((10^5^ cells per 0.3 MF-mg) and thinning the membrane thickness (0.25 mm) to allow penetration into the interior. The red circles in Fig. 5d show the results obtained when the cells were immobilized under these conditions. By using this operation condition, cells could be immobilized further inside of the MF, and a high density of 9.5 × 10^5^ cells MF-fragment^−1^ (3.2 × 10^9^ cells g^−1^) could be achieved, which corresponds to (3.2 × 10^7^ cells ml^−1^) because the volumes of the MFs (0.25 mm) including cells after 16 days of culture were 30 μl per MF fragment.

Finally, the continuous production of exosomes was examined using the cells immobilized in the CPG MF fragment (0.60 mm thickness). The immobilized cells were cultured every 2 days (8 times) in 0.5 ml of ExoFBS-DMEM medium per MF fragment, then subsequently cultured every day (8 times) in 0.25 ml of medium per MF fragment, and finally subsequently cultured twice a day (16 h and 8 h) in 0.125 ml of medium per MF fragment. Figure 5e shows the exosome productivity. The productivity averages of exosomes in 0.5, 0.25, and 0.125 ml of medium per 4 mg MF of fragments were 1.9 × 10^10^, 3.6 × 10^10^ and 7.2 × 10^10^ exosome particles ml ^−1^, suggesting that the higher the cell density per medium, the higher the productivity of exosomes. Additionally, these values were much higher than those of the gelatin-coated CA MF (7.4 × 10^9^ exosome particles ml^−1^ per 2 days). In summary, we confirmed that CPG creates a new type of MF showing superior swelling and cell adhesion properties for obtaining high cell immobilization density and high exosome productivity.

## Discussion

Immobilizing carriers for continuous animal cell culture must be inexpensive, non-water soluble, and have low degradability. Although hydrophobic polymers generally exhibit these characteristics, MFs can hardly take cell suspensions inside because of their hydrophobic surface; therefore, the large surface area inside the MF is barely utilized as scaffolding. This is one of the reasons why MFs have not been used for high-density immobilized cell cultures. Thus, we attempted to develop a new type of MF into which cells can rapidly penetrate deep areas by adding swelling properties and adhere because of its high adhesive properties.

First, we examined the hydrophilicity of MFs and their uptake of water into cells; MFs made of CA, PK, and EVOH were highly hydrophilic and had water retention properties, could take up water in the MFs (Fig. 2), and cells could rapidly be absorbed when the cell suspension was added (Fig. 3). Because these water-insoluble polymers can take up little water in the shapes of particles and films, the acquisition of highly hydrophilic and water-retention properties is thought to be due to the formation of a hydrophilic 3-d structure inside the MFs. In the cell immobilization experiments, PLA, PK, and EVA were superior in cell adhesion for flat films, whereas the NFs of PLA and EVOH were superior in immobilizing cells and gave different results (Fig. 3). Thus, we discovered an interesting phenomenon in which hydrophobic polymers can acquire new properties that differ from their original properties by forming a three-dimensional MF structure.

Second, we produced a novel MF made of blended polymers (CPG) based on the results of the GEL coating experiments (Fig. 4) and swelling experiments (Figs. 2 and 3). This combination can rapidly take cell suspensions into the MF because CA has the swelling property and the flexibility of PK, and immobilization was quickly completed (8 h) without loss of the initially added cells because all cells quickly binding to the MF owing to GEL’s strong cell adhesive capacity (Fig. 5). In conventional methods, such as porous microcarriers, films, and hollow fibers (Ohshima et al. 1999; Looby and Griffiths 1990), many of the initially added cells are lost without immobilization because they do not enter the carriers, which is a major problem. Even when using polymeric MFs or gelatin-coated MFs, > 90% of the cells were lost during immobilization (Figs. 3d and 4), suggesting that the CPG MF has a superior function not found in conventional immobilization carriers. Generally, differentiated normal somatic cells divide very slowly, and cell division in elderly people occurs only a few times due to short telomeres (Shiomi et al. 2024). When such cells are used for immobilization, it is extremely important to be able to immobilize them quickly without loss, suggesting that that CPG MF will be of high value in the future as an immobilization carrier and a cell sheet for the regeneration.

Another notable feature of CPG MFs is their ability to immobilize high-density cells. CPG MF fragments (0.25 mm thickness) could bind up to 3.2 × 10^6^ cells MF-mg^−1^ (3.2 × 10^7^ cells MF-ml^−1^). Several high-density immobilization carriers, including porous carriers and microcarriers, have been reported, such as the immobilization on porous polyvinyl formal resins at 8. 6 × 10^6^ cells PVF-ml^−1^ (Ohshima et al. 1999), 1 × 10^7^ cells ml^−1^ on porous carriers (Looby and Griffiths 1990), and 5 × 10^4^ cells mg^−1^ on the microcarrier Cytodex- 1 (Ferrari et al. 2012). As the density of cells immobilized on the CPG MF is much higher than that of cells immobilized using conventional methods, it is an effective carrier for mass culture.

Moreover, the productivity of exosomes depended on the number of cells per medium (Fig. 5e), and the productivity of exosomes reached a high level by packing cells immobilized on CPG-based MF at high density. Exosomes secreted by vascular endothelial cells are known to prevent osteoporosis (Wang et al. 2024) and improve myocardial infarction (Davidson et al. 2018; Jia and Sowers 2020; Li et al. 2023), and are expected to be therapeutic agents. However, the amount of exosomes secreted by vascular endothelial cells is much lower than that secreted by cancer cells; for example, HUVEC vascular endothelial cells secreted only 4–5 × 10^8^ particles medium-ml^−1^ (Davidson et al. 2018) and the productivity was only 1–1.5 × 10^9^ particles medium-ml^−1^ per 4 days when exosomes were produced in xeno-free medium, a general serum medium, using TKD2 cells grown in a culture dish (results not shown). In contrast, TKD2 vascular endothelial cells immobilized with CPG MF fragments were able to continue producing exosomes for a long time with very high productivity, up to 7 × 10^10^ exosome particles ml^−1^ per 8 or 16 h, by continuous batch culture (Fig. 5e), suggesting that CPG MF can be applied to the mass production of exosomes.

The limitations of this study were as follows. Because it focused on the swelling and cell adhesion properties of MFs, the optimal conditions for the number of cells to be supplied and the thickness and diameter of the MF sheet for immobilizing cells were not deeply studied. The optimal combination of these three polymers and the influence of proteins other than gelatin are unknown. Moreover, it has been noted that exosome production is related to the packing density of cells (Dawson and Weaver 2023), and the relationship between the packing density of cells immobilized on MFs and exosome production needs to be clarified in the future. We are currently studying the efficient production of exosomes using human mesenchymal stem cells immobilized in MFs, and these issues will be reported in our next paper.

## Conclusion

In this study, we aimed to develop MFs suitable for the high-density immobilization of cells, which excelled at absorbing cell suspensions, binding cells, and could rapidly immobilize the supplied cells without loss, suggesting that it is a completely different and efficient method compared to conventional immobilization methods. Moreover, the CPG-based MF was capable of immobilizing high densities of cells and the rapid continuous production of exosomes. Therefore, CPG-based MFs are expected to have a wide range of future applications, including exosome production from animal cells.

## Data Availability

The data supporting the findings of this study are available from the corresponding author upon reasonable request.
